# Supplementary material for: Spinal cord injury and risk of overall and type specific cardiovascular diseases: A meta-analysis
Source: PLoS One. 2024 Oct 28;19(10):e0311572. doi: 10.1371/journal.pone.0311572 (PMC11515965; doi:10.1371/journal.pone.0311572)
Supplement: S1 File — (DOC) [file pone.0311572.s001.doc]

**Supplementary**

**Table S1~S3: Details of the Literature Search Strategy**

(1) PubMed (April 17, 2024)

| No. | Query | Results |
| --- | --- | --- |
| 1 | "Spinal Cord Injuries"[Mesh] | 56,986 |
| 2 | (((((Spinal Cord Trauma*[Title/Abstract]) OR (Traumatic Myelopath*[Title/Abstract])) OR (Spinal Cord Injur*[Title/Abstract])) OR (Spinal Cord Transection*[Title/Abstract])) OR (Spinal Cord Laceration*[Title/Abstract])) OR (Spinal Cord Contusion*[Title/Abstract]) | 51,382 |
| 3 | ("Spinal Cord Injuries"[Mesh]) OR ((((((Spinal Cord Trauma*[Title/Abstract]) OR (Traumatic Myelopath*[Title/Abstract])) OR (Spinal Cord Injur*[Title/Abstract])) OR (Spinal Cord Transection*[Title/Abstract])) OR (Spinal Cord Laceration*[Title/Abstract])) OR (Spinal Cord Contusion*[Title/Abstract])) | 73,194 |
| 4 | ("Cardiovascular Diseases"[Mesh]) OR "Heart Diseases"[Mesh] | 2,779,944 |
| 5 | (((((((((((Cardiovascular[Title/Abstract]) OR (Cardiac Event*[Title/Abstract])) OR (Heart Disease*[Title/Abstract])) OR (Cardiac Disease*[Title/Abstract])) OR (Cardiac Disorder*[Title/Abstract])) OR (Heart Disorder*[Title/Abstract])) OR (coronary artery disease*[Title/Abstract])) OR (myocardial infarction[Title/Abstract])) OR (heart failure[Title/Abstract])) OR (angina pectoris[Title/Abstract])) OR (stroke[Title/Abstract])) OR (hypertension[Title/Abstract]) | 1,724,821 |
| 6 | (("Cardiovascular Diseases"[Mesh]) OR "Heart Diseases"[Mesh]) OR ((((((((((((Cardiovascular[Title/Abstract]) OR (Cardiac Event*[Title/Abstract])) OR (Heart Disease*[Title/Abstract])) OR (Cardiac Disease*[Title/Abstract])) OR (Cardiac Disorder*[Title/Abstract])) OR (Heart Disorder*[Title/Abstract])) OR (coronary artery disease*[Title/Abstract])) OR (myocardial infarction[Title/Abstract])) OR (heart failure[Title/Abstract])) OR (angina pectoris[Title/Abstract])) OR (stroke[Title/Abstract])) OR (hypertension[Title/Abstract])) | 3,400,760 |
| 7 | (("Spinal Cord Injuries"[Mesh]) OR ((((((Spinal Cord Trauma*[Title/Abstract]) OR (Traumatic Myelopath*[Title/Abstract])) OR (Spinal Cord Injur*[Title/Abstract])) OR (Spinal Cord Transection*[Title/Abstract])) OR (Spinal Cord Laceration*[Title/Abstract])) OR (Spinal Cord Contusion*[Title/Abstract]))) AND ((("Cardiovascular Diseases"[Mesh]) OR "Heart Diseases"[Mesh]) OR ((((((((((((Cardiovascular[Title/Abstract]) OR (Cardiac Event*[Title/Abstract])) OR (Heart Disease*[Title/Abstract])) OR (Cardiac Disease*[Title/Abstract])) OR (Cardiac Disorder*[Title/Abstract])) OR (Heart Disorder*[Title/Abstract])) OR (coronary artery disease*[Title/Abstract])) OR (myocardial infarction[Title/Abstract])) OR (heart failure[Title/Abstract])) OR (angina pectoris[Title/Abstract])) OR (stroke[Title/Abstract])) OR (hypertension[Title/Abstract]))) | 7,584 |
| 8 | "Risk"[Mesh] | 1,407,464 |
| 9 | (risk [Title/Abstract]) OR ("Risk"[Mesh]) | 3,420,923 |
| 10 | ((("Spinal Cord Injuries"[Mesh]) OR ((((((Spinal Cord Trauma*[Title/Abstract]) OR (Traumatic Myelopath*[Title/Abstract])) OR (Spinal Cord Injur*[Title/Abstract])) OR (Spinal Cord Transection*[Title/Abstract])) OR (Spinal Cord Laceration*[Title/Abstract])) OR (Spinal Cord Contusion*[Title/Abstract]))) AND ((("Cardiovascular Diseases"[Mesh]) OR "Heart Diseases"[Mesh]) OR ((((((((((((Cardiovascular[Title/Abstract]) OR (Cardiac Event*[Title/Abstract])) OR (Heart Disease*[Title/Abstract])) OR (Cardiac Disease*[Title/Abstract])) OR (Cardiac Disorder*[Title/Abstract])) OR (Heart Disorder*[Title/Abstract])) OR (coronary artery disease*[Title/Abstract])) OR (myocardial infarction[Title/Abstract])) OR (heart failure[Title/Abstract])) OR (angina pectoris[Title/Abstract])) OR (stroke[Title/Abstract])) OR (hypertension[Title/Abstract])))) AND ((risk[Title/Abstract]) OR ("Risk"[Mesh])) | 1,412 |

(2) Embase (April 17, 2024)

| No. | Query | Results |
| --- | --- | --- |
| #13 | #9 AND #12 | 3,746 |
| #12 | #10 OR #11 | 5,111,938 |
| #11 | 'risk':ab,ti | 4,168,501 |
| #10 | 'risk'/exp | 3,214,853 |
| #9 | #3 AND #8 | 19,686 |
| #8 | #6 OR #7 | 6,025,091 |
| #7 | 'cardiovascular':ab,ti OR 'cardiac event*':ab,ti OR 'heart disease*':ab,ti OR 'cardiac disease*':ab,ti OR 'cardiac disorder*':ab,ti OR 'heart disorder*':ab,ti OR 'coronary artery disease*':ab,ti OR 'myocardial infarction':ab,ti OR 'heart failure':ab,ti OR 'angina pectoris':ab,ti OR stroke:ab,ti OR hypertension:ab,ti | 2,527,472 |
| #6 | #4 OR #5 | 5,642,049 |
| #5 | 'heart disease'/exp | 2,511,544 |
| #4 | 'cardiovascular disease'/exp | 5,642,049 |
| #3 | #1 OR #2 | 103,850 |
| #2 | 'spinal cord injury':ab,ti OR 'traumatic myelopath*':ab,ti OR 'spinal cord transection*':ab,ti OR 'spinal cord laceration*':ab,ti OR 'spinal cord contusion*':ab,ti | 58,180 |
| #1 | 'spinal cord injury'/exp | 97,187 |

(3) Cochrane Library (April 17, 2024)

| **Search** | **Query** | **Items found** |
| --- | --- | --- |
| #1 | MeSH descriptor: [Spinal Cord Injuries] explode all trees | 2,453 |
| #2 | (Spinal Cord Trauma*):ti,ab,kw OR (Traumatic Myelopath*):ti,ab,kw OR (Spinal Cord Injur*):ti,ab,kw OR (Spinal Cord Transection*):ti,ab,kw OR (Spinal Cord Laceration*):ti,ab,kw | 4,956 |
| #3 | (Spinal Cord Contusion*):ti,ab,kw | 35 |
| #4 | #1 OR #2 OR #3 | 5,090 |
| #5 | MeSH descriptor: [Cardiovascular Diseases] explode all trees | 155,445 |
| #6 | MeSH descriptor: [Heart Diseases] explode all trees | 74,093 |
| #7 | (Cardiovascular):ti,ab,kw OR (Cardiac Event*):ti,ab,kw OR (Heart Disease*):ti,ab,kw OR (Cardiac Disease*):ti,ab,kw OR (Cardiac Disorder*):ti,ab,kw | 158,275 |
| #8 | (Heart Disorder*):ti,ab,kw OR (coronary artery disease*):ti,ab,kw OR (myocardial infarction):ti,ab,kw OR (heart failure):ti,ab,kw OR (angina pectoris):ti,ab,kw | 108,964 |
| #9 | (stroke):ti,ab,kw OR (hypertension):ti,ab,kw | 146,080 |
| #10 | #5 OR #6 OR #7 OR #8 OR #9 | 343,269 |
| #11 | MeSH descriptor: [Risk] explode all trees | 56,326 |
| #12 | (risk):ti,ab,kw | 313,112 |
| #13 | #11 OR #12 | 317,018 |
| #14 | #4 AND #10 AND #13 | 199 |

**Table S4 Literature to be excluded after reading the full text**

| **Tittle** | **Exclusion reasons** |
| --- | --- |
| Cardiovascular and metabolic complications of spinal cord injury: Findings from a national population health study(1) | Review or Conference Abstract |
| Cardiovascular disease in spinal cord injury: an overview of prevalence, risk, evaluation, and management(2) | Review or Conference Abstract |
| Identification and Management of Cardiometabolic Risk after Spinal Cord Injury: Clinical Practice Guideline for Health Care Providers(3) | Review or Conference Abstract |
| Incidence of Ischemic Stroke in US Veterans Living with Spinal Cord Injury: Preliminary Data(4) | Review or Conference Abstract |
| Presentation of acute myocardial infarction in veterans with spinal cord injury(5) | Review or Conference Abstract |
| Spinal cord injury increases risk for coronary artery disease as determined by coronary artery calcification score(6) | Review or Conference Abstract |
| The Diagnosis and Management of Cardiometabolic Risk and Cardiometabolic Syndrome after Spinal Cord Injury(7) | Review or Conference Abstract |
| A prospective study of health behaviors and risk of all-cause and cause-specific mortality after spinal cord injury(8) | No interested outcomes |
| Cardiovascular Risk Factors Among Older Adults With Long-Term Spinal Cord Injury(9) | No interested outcomes |
| Cardiovascular Risk Markers in Patients with Spinal Cord Injury: The Austrian Wheelchair Dancer Study(10) | No interested outcomes |
| Coronary and carotid imaging of atherosclerosis and contributing factors in middle-aged people with long-term cervical and upper thoracic spinal cord injuries(11) | No interested outcomes |
| Evaluation of cardiovascular disease risk in individuals with chronic spinal cord injury(12) | No interested outcomes |
| Increased risk of acute pancreatitis in persons with spinal cord injury: a population-based, propensity score-matched longitudinal follow-up study(13) | No interested outcomes |
| Increased risk of sensorineural hearing loss in patients with spinal cord injury: a nationwide longitudinal follow-up study(14) | No interested outcomes |
| Respiratory complications during initial rehabilitation and survival following spinal cord injury in Sweden: a retrospective study(15) | No interested outcomes |
| Risk Factors for Pulmonary Infection and Nursing Interventions Post-Tracheostomy in Patients with Spinal Cord Injury(16) | No interested outcomes |
| Relationships between cardiovascular disease risk, neuropathic pain, mental health, and autonomic function in chronic spinal cord injury(17) | Data cannot be extracted |

**Figure S1** Sensitivity analysis of the outcomes of overall types of CVD events

**References:**

1. Cragg JJ, Noonan VK, Dvorak M, Krassioukov A, Borisoff J. Cardiovascular and metabolic complications of spinal cord injury: Findings from a national population health study. J Neurol Sci. 2013;333:e611. http://doi.org/10.1016/j.jns.2013.07.2131

2. Myers J, Lee M, Kiratli J. Cardiovascular disease in spinal cord injury: an overview of prevalence, risk, evaluation, and management. Am J Phys Med Rehab. 2007;86(2):142-52. http://doi.org/10.1097/PHM.0b013e31802f0247

3. Nash MS, Groah SL, Gater DJ, Dyson-Hudson TA, Lieberman JA, Myers J, et al. Identification and Management of Cardiometabolic Risk after Spinal Cord Injury: Clinical Practice Guideline for Health Care Providers. Top Spinal Cord Inj. 2018;24(4):379-423. http://doi.org/10.1310/sci2404-379

4. Willenberg R, Smith B, Stroupe K, Sippel J, Huo Z, Sabharwal S. Incidence of Ischemic Stroke in US Veterans Living with Spinal Cord Injury: Preliminary Data. Neurology. 2023;100(17). http://doi.org/10.1212/WNL.0000000000202615

5. Sabharwal S, Woods P, Stolzmann K, Sharma GVRK, Smith B, Baker E. Presentation of acute myocardial infarction in veterans with spinal cord injury. J Spinal Cord Med. 2013;36(5):516-7. http://doi.org/10.1179/1079026813Z.000000000206

6. McKenna C, Cirnigliaro C, LaFountaine M, Swonger K, Kirshblum S, Spungen AM, et al. Spinal cord injury increases risk for coronary artery disease as determined by coronary artery calcification score. PM and R. 2015;7(9):S97.

7. Farkas GJ, Burton AM, McMillan DW, Sneij A, Gater DJ. The Diagnosis and Management of Cardiometabolic Risk and Cardiometabolic Syndrome after Spinal Cord Injury. J Pers Med. 2022;12(7). http://doi.org/10.3390/jpm12071088

8. DiPiro ND, Cao Y, Krause JS. A prospective study of health behaviors and risk of all-cause and cause-specific mortality after spinal cord injury. Spinal Cord. 2019;57(11):933-41. http://doi.org/10.1038/s41393-019-0298-9

9. Jörgensen S, Hill M, Lexell J. Cardiovascular Risk Factors Among Older Adults With Long-Term Spinal Cord Injury. Pm&R. 2019;11(1):8-16. http://doi.org/10.1016/j.pmrj.2018.06.008

10. Mayr B, Felber M, Frey V, Renz N, Schwenker K, Trinka E, et al. Cardiovascular Risk Markers in Patients with Spinal Cord Injury: The Austrian Wheelchair Dancer Study. Eur J Prev Cardiol. 2023. http://doi.org/10.1093/eurjpc/zwad128

11. Hill M, Jörgensen S, Engström G, Persson M, Lexell J. Coronary and carotid imaging of atherosclerosis and contributing factors in middle-aged people with long-term cervical and upper thoracic spinal cord injuries. Pm&R. 2024;16(3):250-9. http://doi.org/10.1002/pmrj.13043

12. Dorton MC, Lucci VM, de Groot S, Loughin TM, Cragg JJ, Kramer JK, et al. Evaluation of cardiovascular disease risk in individuals with chronic spinal cord injury. Spinal Cord. 2021;59(7):716-29. http://doi.org/10.1038/s41393-020-00566-5

13. Ho WT, Yeh KC, Pan SL. Increased risk of acute pancreatitis in persons with spinal cord injury: a population-based, propensity score-matched longitudinal follow-up study. Spinal Cord. 2021;59(11):1170-6. http://doi.org/10.1038/s41393-021-00643-3

14. Yang SM, Yeh KC, Pan SL. Increased risk of sensorineural hearing loss in patients with spinal cord injury: a nationwide longitudinal follow-up study. Spinal Cord. 2021;59(11):1200-5. http://doi.org/10.1038/s41393-021-00697-3

15. Josefson C, Rekand T, Lundgren-Nilsson Å, Sunnerhagen KS. Respiratory complications during initial rehabilitation and survival following spinal cord injury in Sweden: a retrospective study. Spinal Cord. 2021;59(6):659-64. http://doi.org/10.1038/s41393-020-00549-6

16. Lv Q, Zhang X, Guo K, Hu D, Deng Z. Risk Factors for Pulmonary Infection and Nursing Interventions Post-Tracheostomy in Patients with Spinal Cord Injury. Infect Drug Resist. 2023;16:6691-701. http://doi.org/10.2147/IDR.S418894

17. Dorton MC, Kramer JK, de Groot S, Post MWM, Claydon VE. Relationships between cardiovascular disease risk, neuropathic pain, mental health, and autonomic function in chronic spinal cord injury. Spinal Cord. 2023;61(10):548-55. http://doi.org/10.1038/s41393-023-00933-y
